# Distributed Medical Image Analysis and Diagnosis through Crowd-Sourced Games: A Malaria Case Study

**DOI:** 10.1371/journal.pone.0037245

**Published:** 2012-05-11

**Authors:** Sam Mavandadi, Stoyan Dimitrov, Steve Feng, Frank Yu, Uzair Sikora, Oguzhan Yaglidere, Swati Padmanabhan, Karin Nielsen, Aydogan Ozcan

**Affiliations:** 1 Electrical Engineering Department, University of California Los Angeles, Los Angeles, California, United States of America; 2 Division of Infectious Diseases, Department of Pediatrics, School of Medicine, University of California Los Angeles, Los Angeles, California, United States of America; 3 Bioengineering Department, University of California Los Angeles, Los Angeles, California, United States of America; 4 California NanoSystems Institute, University of California Los Angeles, Los Angeles, California, United States of America; 5 Department of Surgery, School of Medicine, University of California Los Angeles, Los Angeles, California, United States of America; Centro de Pesquisa Rene Rachou/Fundação Oswaldo Cruz (Fiocruz-Minas), Brazil

## Abstract

In this work we investigate whether the innate visual recognition and learning capabilities of untrained humans can be used in conducting reliable microscopic analysis of biomedical samples toward diagnosis. For this purpose, we designed entertaining digital games that are interfaced with artificial learning and processing back-ends to demonstrate that in the case of binary medical diagnostics decisions (e.g., infected vs. uninfected), with the use of crowd-sourced games it is possible to approach the accuracy of medical experts in making such diagnoses. Specifically, using non-expert gamers we report diagnosis of malaria infected red blood cells with an accuracy that is within 1.25% of the diagnostics decisions made by a trained medical professional.

## Introduction

Crowd-sourcing is an emerging concept that has attracted significant attention in recent years as a strategy for solving computationally expensive and difficult problems [1]–[6]. In this computing paradigm, pieces of difficult computational problems are distributed to a large number of individuals. Each participant completes one piece of the computational puzzle, sending the results back to a central system where they are all combined together to formulate the overall solution to the original problem. In this context, crowd-sourcing is often used as a solution to various pattern-recognition and analysis tasks that may take computers long times to solve. One of the underlying assumptions of such an approach is that humans are better than machines at certain computational and pattern recognition tasks.

There has been much work in the general field of ‘gaming’ as a method for crowd-sourcing of computational tasks [1], [7]–[13]. Digital games have been used as effective means to engage an individual's attention to computational tasks of interest. If a pattern-recognition task can be embedded as part of an engaging game, then a gamer may help in solving this task together with other gamers. Recently a number of gaming platforms have been created to tackle problems in e.g., biology and medical sciences, allowing non-experts to take part in solving such problems. FoldIt [7]–[8], as an example, is a game in which players attempt to digitally simulate folding of various proteins, helping researchers to achieve better predictions about protein structures. EteRNA [9] is another game, which likewise makes use of crowds to get a better understanding of RNA folding.

**Figure 1:**
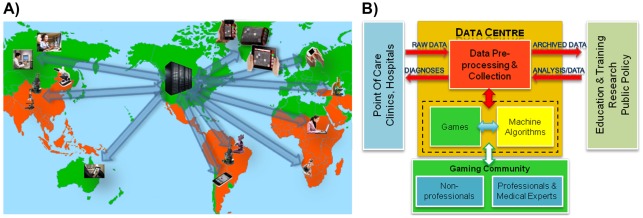
Proposed platform. **A**) Biomedical data (e.g., images of thin blood smear samples) from individual light microscopes all around the world are transmitted to data centres where they are pre-processed and digitally distributed among gamers, which in turn diagnose and transmit their responses back. These individual results of the gamers are then fused toward a final diagnosis, the result of which is transmitted back to the point-of-care or the clinic/hospital. In the map above, orange-coloured regions show locations where risk of contraction of malaria still exists. **B**) Block diagram of the presented platform.

In this work, we take a similar strategy and demonstrate a platform to use digital gaming and machine learning to crowd-source the analysis of optical microscopy images of biomedical specimens through engaging the interest of human game players (i.e., gamers). The primary goal of this methodology is to accurately diagnose medical conditions, approaching the overall accuracy of medical experts, while only using non-expert gamers (see Figure 1). The same method can also function as a telemedicine platform, where trained medical experts could be made part of our gamer crowd through various incentives.

**Figure 2:**
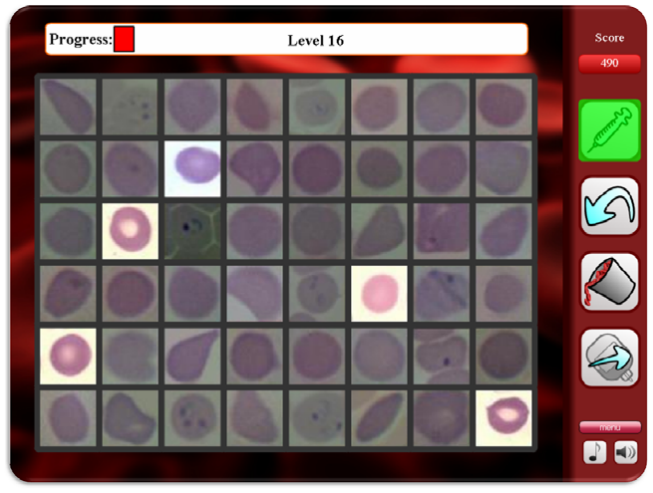
Malaria Diagnosis Game Interface: the gamer can use the syringe tool to “kill” infected RBCs or use the blood bank button to “bank/collect” all the healthy RBCs.

In general there is much detail and subtlety associated with medical images, and therefore accurate analysis and interpretation of such images often become tedious and time consuming, even for highly trained professionals. Crowd-sourcing of microscopic analysis and related diagnosis through gaming is rather timely in several ways. First, with rapid advances in mobile telecommunication and internet technologies such as mobile-phones, tablet PCs, etc., we have hundreds of millions of active users and potential gamers in the cloud that are all connected to a global network. In addition to this massive crowd volume, over the last few years, there has been a significant effort to create cost-effective, compact and lightweight microscope designs such that even mobile-phones could be converted into microscopic analysis tools [14]–[21]. Similar to the development of the PC, this is a very important development since it could enable wide-spread use of optical microscopy globally, with several orders of magnitude increase in the number of microscope users over the next decade. As will be further discussed in this manuscript, these recent advances make it feasible to create a self-learning platform that leverages crowd-sourcing and gaming concepts to conduct accurate and sensitive microscopic analysis of biomedical specimens.

**Figure 3:**
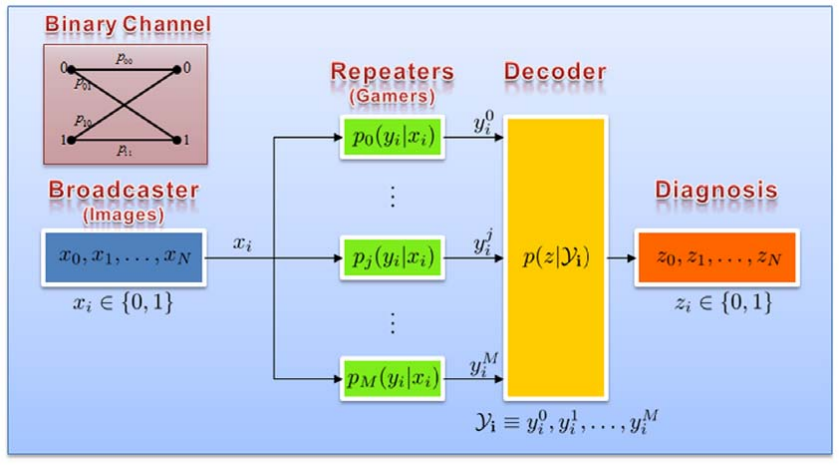
Overview of the gaming analysis framework. The images are treated as a sequence of binary values that are broadcast by the server. The gamers are effectively noisy repeaters that in the most ideal case output the correct symbol for the inputs that they receive. Each repeater transmits its own noisy version of the same input symbol to a decoder. The decoder combines all the received repeater outputs and decodes a final output z_i_, which ideally will be the correct label/diagnosis for the input images. The repeaters can be modelled as Binary Communication Channels (top-left). *p_ij_* corresponds to the probability of receiving symbol *j* when in fact symbol *i* was transmitted.

**Table 1:** Summary of experimental results for diagnosis of malaria infected red blood cells.

Experiment	Description of Test Images	Gamers	Positive RBCs	Negative RBCs	Control Images	Accuracy	SE	SP	PPV	NPV
1	5055 test RBC images crowd-sourced to human gamers	19	471	4584	1266	99.01%	95.12%	99.41%	94.32%	99.50%
2	5055 test RBC images presented to a boosted set of classifiers, trained on 1266 RBC images	NA	471	4584	1266 (Training Images)	96.26%	69.64%	99.00%	87.70%	96.95%
3	459 low-confidence test images taken from the results of experiment 2	27	274	185	1266	95.42%	97.81%	91.89%	94.70%	96.59%
4	Hybrid diagnosis results using experiments 2 & 3	27	471	4584	1266	98.50%	89.38%	99.43%	94.18%	98.91%
5	7045 test RBC images crowd-sourced to human gamers	20	1549	5496	2349	98.78%	97.81%	99.05%	96.68%	99.38%

We believe that this crowd-sourcing and gaming based micro-analysis platform could in particular be significant for telemedicine applications such that diagnostics decisions can be remotely made without the need for a local medical expert (e.g., a pathologist), especially impacting the medical infrastructure in resource-poor countries. We hypothesise that the gaming community can develop better recognition skills over time for specific medical conditions through a scoring system built in the games to identify such abilities of individuals (some of whom may also be health-care professionals that e.g., are even paid for each image that is diagnosed as part of the game). In addition to providing accurate remote diagnosis, the datasets of biomedical images characterized through this distributed platform can potentially be used as training images for automated machine learning based algorithms that over time self-learn to make reliable diagnosis (Figure 1). As such, through this crowd-sourcing and gaming platform we can create a self-learning integrated network of microscopes and imagers toward intelligent automated biomedical micro-analysis and diagnosis. Once scaled up, this smart network may have a significant impact on e.g., medical, environmental, and biological sciences, among others, through various innovative uses of this network and its constantly expanding database. For instance, by creating large data libraries of various specimens (e.g., microbial communities, parasites, pathology slides, blood/sputum/pap smears etc.) we may be able to dynamically track both temporal and spatial evolution of different pathogens, diseases, or infectious outbreaks and be able to better investigate and identify the cause-effect relationships of these spatiotemporal patterns (see Figure 1).

**Figure 4:**
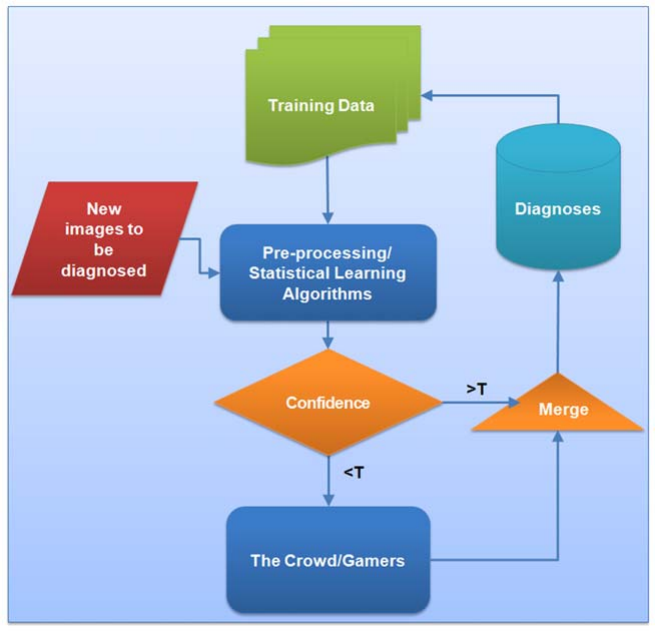
The hybrid (human + machine) diagnostics framework. As new images are generated, they are diagnosed by pre-trained machine learning algorithms. The confidence of these algorithms in their decisions determines whether the images should be passed on to human gamers or not. Once the *difficult-to-diagnose* images for these algorithms are crowd-sourced and are diagnosed by the human gamers, they are merged with the *easy-to-diagnose* images to compute the final diagnostics results. The data is then fed back through the system and added to the training dataset used by the machine learning algorithms. During each cycle, self-learning algorithms will improve as a result of added training data. ‘T’ refers to a threshold value.

**Figure 5:**
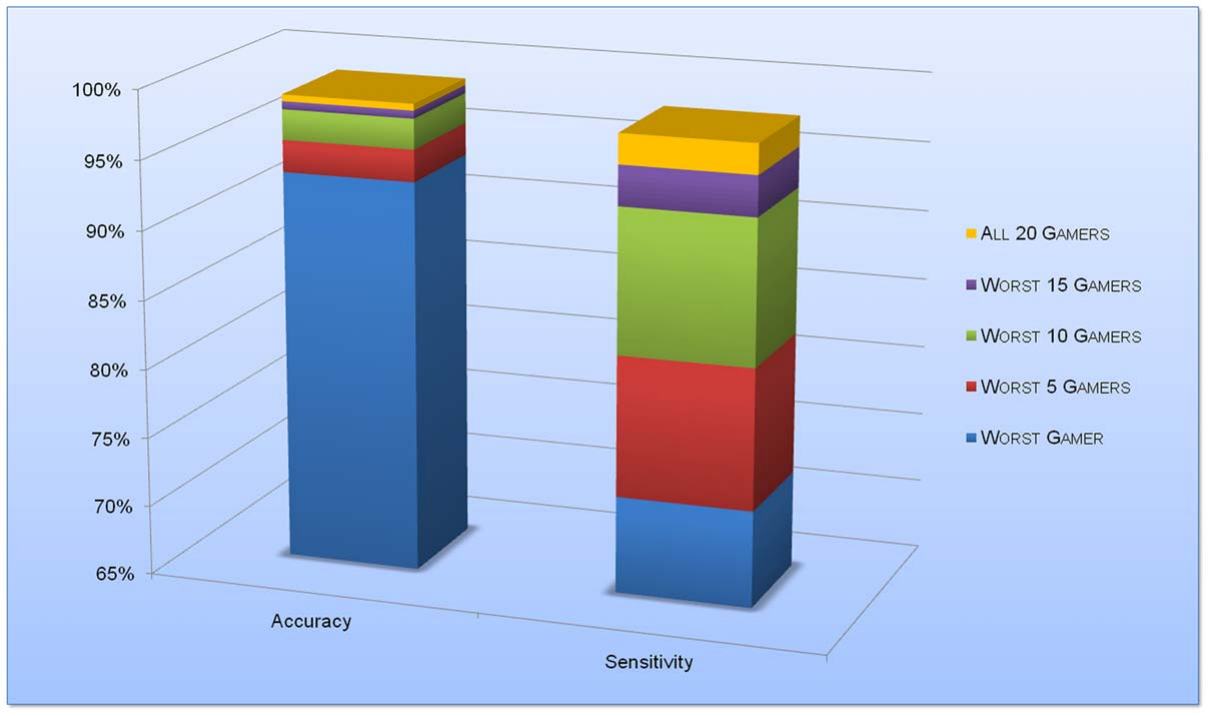
The Crowd Effect: gamer performance results for experiment #5. The plots show the worst case scenarios where the diagnoses from the worst performing players are used to generate an overall diagnosis for each RBC in the game. Note that the specificity (or true negative rate) is always very high for the gamers, and does not improve much as more gamers are added to the mix. However, the sensitivity (or true positive rate) benefits the most as more players are added, and climbs above 95% once 15-gamers form the crowd. The accuracy also increases as more players are added, but since it reflects both the specificity and the sensitivity its increase is not as drastic as that of the sensitivity.

To demonstrate the proof-of-concept of the above outlined framework, we chose ***malaria*** as the medical condition to be diagnosed, and developed a crowd-sourcing and distributed gaming platform that allows individuals from anywhere in the world to assist in identifying malaria infected red blood cells (RBCs) imaged under light microscopes. In addition, we developed an automated algorithm for diagnosing the same images using computer vision, and created a novel hybrid platform for combining human and machine resources toward efficient, accurate and remote diagnosis of malaria.

For this initial demonstration, we chose malaria since it is still a major health problem in many tropical and sub-tropical climates, including much of sub-Saharan Africa (see Figure 1). It is the cause of ∼20% of all childhood deaths in this region, and almost 40% of all hospitalizations in whole of Africa. For diagnosis of malaria, conventional light microscopy remains the gold standard method. A pathologist must typically check between 100 and 300 different field-of-views (FOVs) of a thin blood smear (corresponding to inspection of at least 1,000 individual RBCs) using a light microscope with 100X objective lens before being able to reliably call a thin smear sample negative (i.e. not infected). This, however, is a very time-consuming task and a significant challenge given the large number of cases observed in these resource-poor settings (see Figure 1). Furthermore, approximately 60% of the cases reported in sub-Saharan Africa are actually false-positives,[22] leading to unnecessary treatments and hospitalizations.

For the same purpose of malaria diagnosis in resource poor conditions, rapid diagnostic tests (RDTs) are also being developed to create an alternative to optical microscopy. However, RDTs still have various shortcomings such as: (**i**) being relatively expensive compared to microscopy, costing ∼0.5–2.0 USD per test; (**ii**) existence of insufficient information on their quality and the lack of ability to test their performance in the field, (i.e., the lack of quality controls); (**iii**) poor heat and humidity stability; and (**iv**) mistrust of RDTs by health-care workers and community members (which is technically coupled to issues **i** and **iv** mentioned above) [22]. Therefore, microscopic imaging of blood smears still remains as the gold standard method toward diagnosis of malaria.

There have been prior attempts based on machine vision algorithms to automate the process of malaria diagnosis in Giemsa-stained thin blood smears using optical microscopy images with promising performance results [23]–[27]. However, there are a number of factors that can negatively affect the performance of such algorithms, including variations in blood smear preparation and cell density on the slide, as well as variations in illumination, digital recording conditions, optical aberrations and the lens quality. As a result, these methodologies have not yet been able to find their ways into mainstream malaria diagnostics tools to start replacing manual inspection of blood smears.

The human visual system, however, does not suffer from the above mentioned limitations, and can correctly recognise patterns of parasitic infection even under severe variations in sample preparation, density and imaging/recording conditions. As such, we believe that scaling up accurate, automated, and remote diagnosis of malaria through a crowd-sourcing and gaming platform may achieve significant impact in the developing world through: (**i**) Elimination of the overuse and misuse of anti-malarial drugs, which is quite important for avoiding long-term drug resistance issues; (**ii**) Improved management of non-malaria fevers by allowing malaria to be ruled out, so that patients can receive more appropriate treatment; (**iii**) Much better use of existing funds and reduction in drug stock-outs; and (**iv**) Reduced risks due to long-term side-effects of anti-malarial drugs on patients who do not actually need treatment. Finally, we should also emphasize that the same crowd-sourcing and gaming based micro-analysis and medical diagnosis platform could further scale up for a variety of other biomedical and environmental applications where microscopic images need to be examined by experts.

## Methods

### Game Design

We developed a digital gaming platform through which we allow an unlimited number of gamers from any location in the world to access and diagnose images of human RBCs that are potentially infected with *P. falciparum*. The game (see Figure 2) was implemented to be run both on PCs (using Adobe Flash running on any internet browser such as Internet Explorer, Mozilla Firefox etc.) and on Android-based mobile devices, including mobile-phones as well as tablet PCs. We preferred an open-source operating system for mobile devices such that other game developers could easily contribute to the same crowd-sourcing platform in the future.

Before starting to play the game, each gamer was given a brief online tutorial explaining the rules of the game and how malaria infected RBCs typically look with some example images. After this, each gamer played a training game where s/he was required to successfully complete in order to continue playing the rest of the game. This test game consisted of 261 unique RBC images, where 20 of them were infected. The gamers were required to achieve >99% accuracy in this training game, and in the case of failure, they were asked to re-play it until they achieved 99%. This way all the gamers became familiar with the rules of the game and were briefly trained on the diagnostics task. Note that this training game was required only once at the time when the gamers registered on our platform. Upon registration, a unique user ID was assigned to each gamer and her/his individual diagnostics performance was tracked. Furthermore, this training game provided direct feedback to the players on their performance and their mistakes through a scoring mechanism. Since the labels (i.e., infected cell vs. healthy cell) of all the images were known *a priori* for the purposes of this training game, the player's score was updated throughout the game (i.e., positive score for correct diagnosis, and negative score for incorrect diagnosis). It is important to note that there exists a large body of work on educational games [28]–[29]. However, given that our focus was not to educate the players, and in fact it was to demonstrate the quality of diagnostic results that can be achieved through untrained (non-expert) individuals, this initial test/training game was designed in a simple repetitive fashion.

As the gamer goes through the game, s/he is presented with multiple frames of RBC images. The gamer has the option of using a “*syringe”* tool to “*kill*” the *infected* cells one by one, or use a “*collect-all*” tool to designate all the remaining cells in the current frame as “*healthy*”, which significantly speeds up the cell diagnosis process since most of the RBCs are healthy. Within each frame, there are a certain number of cells whose labels (infected or healthy) are known to the game, but unknown to the gamers. These *control* cell images allow us to dynamically estimate the performance of the gamers (in terms of correct and incorrect diagnosis) as they go through each frame and also help us assign a score for every frame that they pass through (Note that this is different in the training game where all the images are effectively control images). Once a frame is completed, a score is assigned based on the performance of the gamer only on the control images. These control images (roughly 20% of all the images) along with the scoring system allow the game to provide some feedback to the gamer on their performance such that as the gamers continue to play, they can improve their diagnostics performance. The images and their order of appearance were identical among different gamers, thus allowing us to make a fair comparison among their relative performances.

### Image Database

To build a malaria infected RBC database, we used thin blood smear slides that contained mono-layers of cultured human RBCs which were infected by *Plasmodium falciparum (P. falciparum)* forming the source for our image dataset (refer to Reference [17] for further details). These malaria slides were then scanned with a bright-field optical microscope using a 100X oil-immersion objective lens (numerical aperture: 1.25). At each FOV, the captured RBC images were passed on to an infectious disease expert for identification of *P. falciparum* signatures and digital labeling of each RBC image (positive vs. negative). This process generated a dataset of 7116 unique RBC images, with 1603 of them infected by the malaria parasite. To form the set of images to be used in our games, each individual RBC image was cropped and resized to fixed dimensions of 50×50 pixels. To further increase the total number of images and their diversity (in terms of sample preparation, density and imaging conditions), we also used a set of images provided by the Center for Disease Control (CDC), yielding an addition of 118 infected and 595 uninfected RBC images. With this, we had a total of 7829 characterized human RBC images, with 1721 of them infected with *P. falciparum*, forming our *ground truth database* for evaluating our crowd-sourcing, gaming, and machine-vision based diagnostics platform. Please refer to Text S1 for further details and Figure S4 for sample images. No IRB approval was required since the digital red blood cell images that we used in our work were not linked to any patient data or diagnosis and were digitally created and shared for microscopic training purposes.

### Diagnostic Analysis

When analysing the game results, we have access to the individual performance parameters and diagnoses (for both the control images and the unknown test images). We fuse the results from all gamers that have completed a particular game and generate a more accurate set of diagnoses for the test RBC images. Given that each RBC image either corresponds to a healthy cell or an infected cell, we can use binary labels to identify them: 0 for healthy and 1 for infected. Recasting our setup as a *communications system*, our server will act as a broadcaster of a binary sequence and each gamer will act as a *noisy Binary Channel* [30], retransmitting the symbols back along with some errors. Therefore, we model the framework of our games as a *noisy communication network* consisting of a broadcast unit, multiple repeaters, and a receiver/decoder unit for the final diagnosis (see Figure 3). In the ideal scenario, the repeaters (i.e. the gamers) would simply receive a set of incoming symbols (images to be diagnosed) from the broadcast unit (through various light microscopes located in e.g., point-of-care offices or malaria clinics), and transmit them to the receiver/decoder block, which in turn computes the optimal “correct” label for each individual unknown RBC image using a *Maximum a Posteriori Probability* (*MAP*) approach. In Text S1 (Section II) we provide a theoretical description of how this performance analysis is done and is used to diagnose unknown RBCs based on gamers' responses.

## Results and Discussion

To test the viability of our crowd-sourced gaming-based malaria diagnosis platform, different experiments were run with 31 unique participants (*non-experts*), ranging between the ages of 18 and 40. In total, five different experiments were performed, the results of which are summarized in Table 1.

We initially tested the capability of the presented platform through a game consisting of 5055 images, of which 471 were of infected RBCs and 4584 were of healthy RBCs (see Table 1). Additionally, 1266 (103 positives and 1163 negatives) RBC images were embedded as control images within the same game such that each gamer had to go through 6321 RBC images. The combined accuracy of the gamer diagnoses was 99%, with sensitivity (SE) of 95.1% and specificity (SP) of 99.4%. The positive predictive value (PPV) and negative predictive value (NPV) were also quite high at 94.3% and 99.5% respectively (for definitions of SE, SP, PPV, and NPV refer to Table S1).

In addition to the gaming and the crowd-sourcing platform described earlier, we also developed an automated computer vision-based algorithm to detect the presence of malaria parasites (refer to Text S1–Section III and Figure S1 for details of implementation). In doing so our aim was to ultimately create a hybrid system such that machine vision and human vision can be coupled to each other, creating a more efficient and accurate biomedical diagnostics platform. For this purpose, *independent of the human crowd*, we next tested the automated diagnosis performance of our machine-vision algorithm, which was trained on 1266 RBC images (*same as the control images used in experiment #1*) and was tested on a total of 5055 unique RBC images (471 positives and 4584 negatives – see Table 1). This algorithm was able to achieve an overall accuracy of 96.3%, with SE-SP of 69.6%–99.0%, and PPV-NPV of 87.7%–96.9%. In terms of performance, our gamer crowd did better than this machine algorithm as summarized in Table 1. However, we should note that with an even larger training dataset (containing e.g., >10,000 RBC images) and more advanced classifiers, it may be possible to significantly improve the performance of our automated algorithm. This feat may be achieved through the coupling of statistical learning and crowd-sourcing into a hybrid model as illustrated in Figure 4, where a feedback exists between the gamers and the automated algorithm, yielding an ever-enlarging training dataset as more games are played. This uni-directional feedback loop has the effect of labelling more and more images as training data for the automated algorithm, potentially leaving only the most difficult ones to be labelled by human gamers.

Following this initial comparison between human vision and machine vision for identification of malaria infected RBCs, to assess the viability of the above discussed hybrid diagnosis methodology, we conducted another test (experiments #3 & #4 in Table 1), where among all the RBC images characterized using our machine-vision algorithm, we extracted the ones with a diagnosis confidence level that is less than 30% of the maximum achieved confidence level, i.e. a total of 459 RBC images that were relatively difficult to diagnose were extracted. The training dataset (1266 RBC images that were used to train our machine algorithm, which also served as the control images of experiment #1) were then mixed with these “*difficult-to-diagnose*” 459 RBC images and were used to form a new game that is crowd-sourced to 27 human gamers. This new game (experiment #3) yielded an accuracy of 95.4%, with SE-SP at 97.8%–91.9%, and PPV-NPV at 94.7%–96.6% on these 459 difficult-to-diagnose RBC images. Next, we merged the results from the crowd-sourced game (experiment #3) and our machine algorithm (experiment #2) to arrive at an overall accuracy of 98.5%, with SE-SP of 89.4%–99.4% and PPV-NPV of 94.2%–98.9% (see experiment #4, Table 1). *Thus, in this hybrid case we were able to increase the specificity and positive predictive value by 20% and 7%, respectively, and achieved a performance comparable to that of a completely human-labelled system (experiment #1), but with* ***only 10%*** *of the number of cells actually being labelled by humans*. This significantly increases the efficiency of the presented gaming platform such that the innate visual and pattern-recognition abilities of the human crowd/gamers is put to much better use by only focusing on the ‘difficult-to-diagnose’ images through the hybrid system (Figure 4).

In our next experiment (# 5) we increased the number of infected RBC images in the game by three-fold to simulate a scaled up version of the gaming platform. A total of 7829 unique RBC images were incorporated into the game, of which 784 were taken as control images that were repeatedly inserted into the game for a total of 2349 times. As a result, each gamer would go through 9394 RBC images, a quarter of which (2349) are known control images. Within the remaining 7045 test RBC images, there were 1549 (22%) positive images and 5496 negative images, which were all treated as unknown images to be diagnosed by the human crowd at the single cell level. The same ratio of positive to negative images was also chosen for the control RBC images in the game to eliminate any unfair estimation biases that may result from differing distributions. Completing this game (i.e. experiment # 5) took on average less than one hour for each gamer, and we can see in Table 1 that the accuracy of the overall human crowd (non-professionals) is within ***1.25%*** of the diagnostic decisions made by the infectious disease expert. This experiment yielded an SE of 97.8% and an SP of 99.1%. The PPV was 96.7% and the NPV was 99.4%. The performance results of the individual players and their combined performances are shown in Figures S2 and S3.

Based on experiment #5, Figure 5 summarizes “the effect of the crowd” on diagnosis accuracy and sensitivity, i.e., how the overall performance of the crowd's diagnosis is improved as more gamers are added to the system. We can see significant boosts in the sensitivity (i.e., the true positive rate) as diagnosis results from more gamers are added into the system. This is quite important as one of the major challenges in malaria diagnosis in sub-Saharan Africa is the unacceptably high false-positive rate, reaching ∼60% of the reported cases [22]. Our overall diagnosis accuracy also steadily improves as more gamers are added as shown in Figure 5. This crowd effect may seem like a deviation from the traditional benefits of crowd-sourcing, in that multiple players are inaccurately solving the whole puzzle and then their results are combined to yield a more accurate solution. However, we should also note that cell images from a single blood smear slide can be broken up into multiple batches, where each batch is crowd-sourced to a group of players. In other words, each unique group of players will focus on one common batch of cell images, and in the end the diagnosis results will be combined once at the group level to boost the accuracies for each cell, and again at the slide level to make a correct overall diagnosis per patient. Therefore, the contribution of the crowd is twofold. First, it allows for the analysis problem to be broken up into smaller batches, and second, the analysis of the same batch by multiple individuals from the crowd allows for significantly higher overall diagnosis accuracies.

We should emphasise that throughout the manuscript we discuss diagnosis results for ‘individual’ RBCs, not for patients. In reality, malaria diagnosis using a blood smear sample corresponding to a patient is a relatively easier task compared to single cell diagnosis since a thin blood smear for each patient sample already contains thousands of RBCs on it. Therefore statistical errors in the parasite recognition task could be partially hidden if the diagnostics decisions are made on a per blood-smear slide basis. To better demonstrate the proof of concept of our gaming based crowd-sourcing approach we aimed for the diagnosis of individual RBCs, rather than patients. Since any given patient's blood smear slide will be digitally divided into smaller images (containing e.g., a handful of RBCs per image), and >1,000 RBC images per patient will be distributed to the crowd, we expect much higher levels of accuracy and sensitivity for diagnosis of individual patients. Furthermore, our single-cell-diagnosis-based gaming approach could also be very useful to estimate the parasitemia rate of patients which can be quite important and valuable for monitoring the treatment of malaria patients.

We should also emphasise that the work presented in this paper is a proof of concept and not the complete envisioned system, with potentially thousands of gamers and many patient slides to be diagnosed, which is left as future work. In addition to generating remote biomedical diagnosis through engaging games, the presented platform can serve as an information hub for the global healthcare community as summarized in Figure 1. This digital hub will allow for the creation of very large databases of microscopic images that can be used for e.g., the purposes of training and fine tuning automated computer vision algorithms. It can also serve as an analysis tool for health-care policy makers toward e.g., better management and/or prevention of pandemics.

Next, we would like to briefly discuss regulatory and practical issues that need to be addressed for deployment of the presented gaming and crowd-sourcing-based diagnosis and telemedicine platform. As a potential future expansion of the platform, incentives (e.g., monetary ones) can be used to recruit health-care professionals who are trained and educated to diagnose such biomedical conditions, making them part of our gamer crowd. In such a scenario, one can envision the gaming platform to serve as an intelligent telemedicine backbone that helps the sharing of medical resources through e.g., remote diagnosis and centralised data collection/processing. In other words, it would be a platform whereby the diagnosis can take place by professionals far away from the point-of-care. At the same time, it also enables the resolution of possible conflicting diagnostics decisions among medical experts, potentially improving the diagnostics outcome.

For this potentially highly trained crowd of “professional” gamers, the final decisions made through the crowd can be used for direct treatment of the patient (without the need for regulatory approval). Furthermore, since these are trained medical professionals, the number of gamers assigned to an image that is waiting to be diagnosed can be significantly lower as compared to the case where “non-professional” gamers are assigned to the same image. On the other hand, if an image is diagnosed by entirely non-professional gamers, the result of the diagnosis can still be very useful to reduce the workload of health-care professionals located at point-of-care offices or clinics where the raw images were acquired. In the case of malaria diagnosis, this is especially relevant since the health-care professional is required to look at >1,000 RBC images for accurate diagnosis. Hence even a non-professional crowd's diagnostics decisions could be highly valuable in guiding the local medical expert through the examination of a malaria slide, such that the most relevant RBC images are quickly screened first, eliminating the need for conducting a manual random scan for rare parasite signatures.

Finally, the proposed methodology can be expanded to include a ‘training platform’. Assuming the expansion of this crowd-sourced diagnostics platform and the generation of large image databases with correct diagnostics labels, software can be created to make use of such databases to assist in the training of medical professionals. Through such software, medical students and/or trainees can spend time looking at thousands of images, attempting diagnosis, and getting real-time feedback on their performances. Based on the concepts described in this paper, we also envision this platform to expand to other micro-analysis and diagnostics needs where biomedical images need to be examined by experts.

## Supporting Information

Figure S1 **Local Colour Peak Histograms** (**LCPH**)**.** For every window block, a colour histogram is calculated. The dominant pair of colours is used to compute an index (e.g., with 5 bins, there are a total of 10 different index values). A histogram of all index values is computed and used as part of the feature vector. In addition to colour-based features, we also used a number of more basic image features such as mean, variance, and gradient magnitude histograms to form our final feature vectors.
(TIF)

Figure S2 **Individual performance results for experiment 5 of the Main Text.** A total of 20 individual gamers played this game that consisted of 7045 test cell images with 1549 cells infected and 5496 cells healthy. We use a maximum a posterior probability (MAP) based approach to combine the results of multiple gamers and obtain higher performance levels. In order to show the worst case scenario, the worst *N* performances are combined in each MAP estimation.
(TIF)

Figure S3 **Combined performance of gamers using the described MAP approach.** We observe that if we combine all the available gamer data, we can determine the best gamer relative to others regardless of the ground truth data, lending itself to a scoring and ranking mechanism for gamers.
(TIF)

Figure S4 **Examples of RBC images used in experiments and games.** The images exhibit significantly different illumination conditions, colours and backgrounds, mimicking a real-life scenario where various different optical microscopes located at e.g., point-of-care offices and malaria clinics would be used in our games.
(TIF)

Table S1 **Definition of acronyms used in the manuscript.**
(PDF)

Text S1 **This supporting text describes the mathematical and algorithmic details of the proposed framework.**
(PDF)
