# Supplementary figures and images for: Distributed Medical Image Analysis and Diagnosis through Crowd-Sourced Games: A Malaria Case Study
Source: PLoS One. 2012 May 11;7(5):e37245. doi: 10.1371/journal.pone.0037245 (PMC3350488; doi:10.1371/journal.pone.0037245)

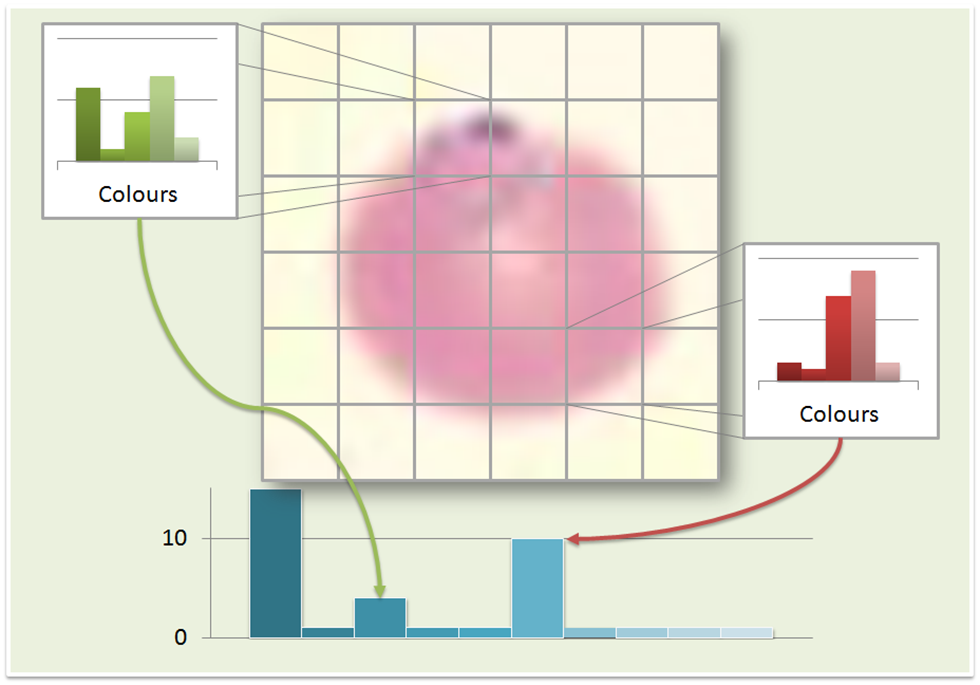

Supplement: Figure S1 — Local Colour Peak Histograms (LCPH). For every window block, a colour histogram is calculated. The dominant pair of colours is used to compute an index (e.g., with 5 bins, there are a total of 10 different index values). A histogram of all index values is computed and used as part of the feature vector. In addition to colour-based features, we also used a number of more basic image features such as mean, variance, and gradient magnitude histograms to form our final feature vectors. (TIF) [file pone.0037245.s001.tif]

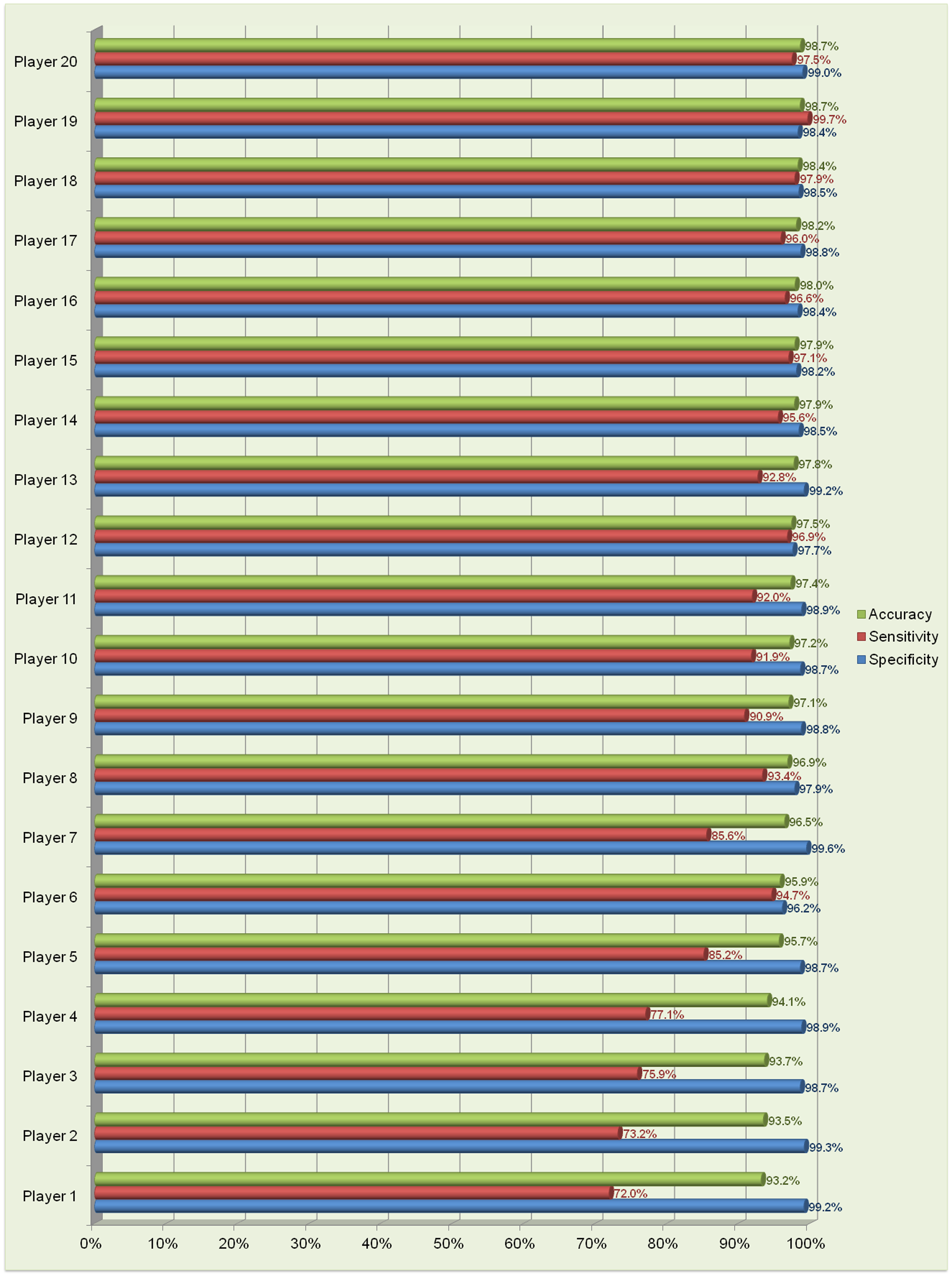

Supplement: Figure S2 — Individual performance results for experiment 5 of the Main Text. A total of 20 individual gamers played this game that consisted of 7045 test cell images with 1549 cells infected and 5496 cells healthy. We use a maximum a posterior probability (MAP) based approach to combine the results of multiple gamers and obtain higher performance levels. In order to show the worst case scenario, the worst N performances are combined in each MAP estimation. (TIF) [file pone.0037245.s002.tif]

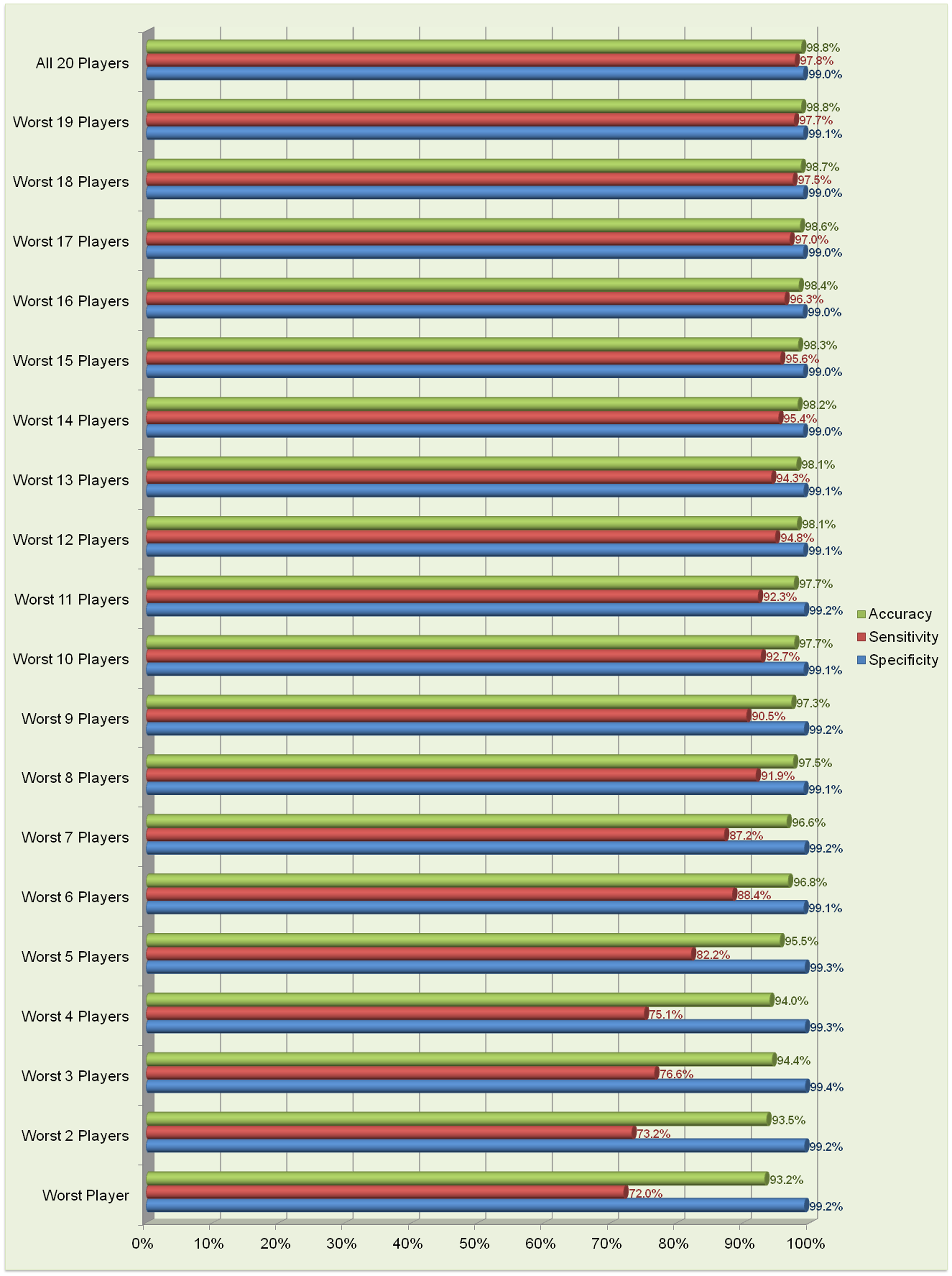

Supplement: Figure S3 — Combined performance of gamers using the described MAP approach. We observe that if we combine all the available gamer data, we can determine the best gamer relative to others regardless of the ground truth data, lending itself to a scoring and ranking mechanism for gamers. (TIF) [file pone.0037245.s003.tif]

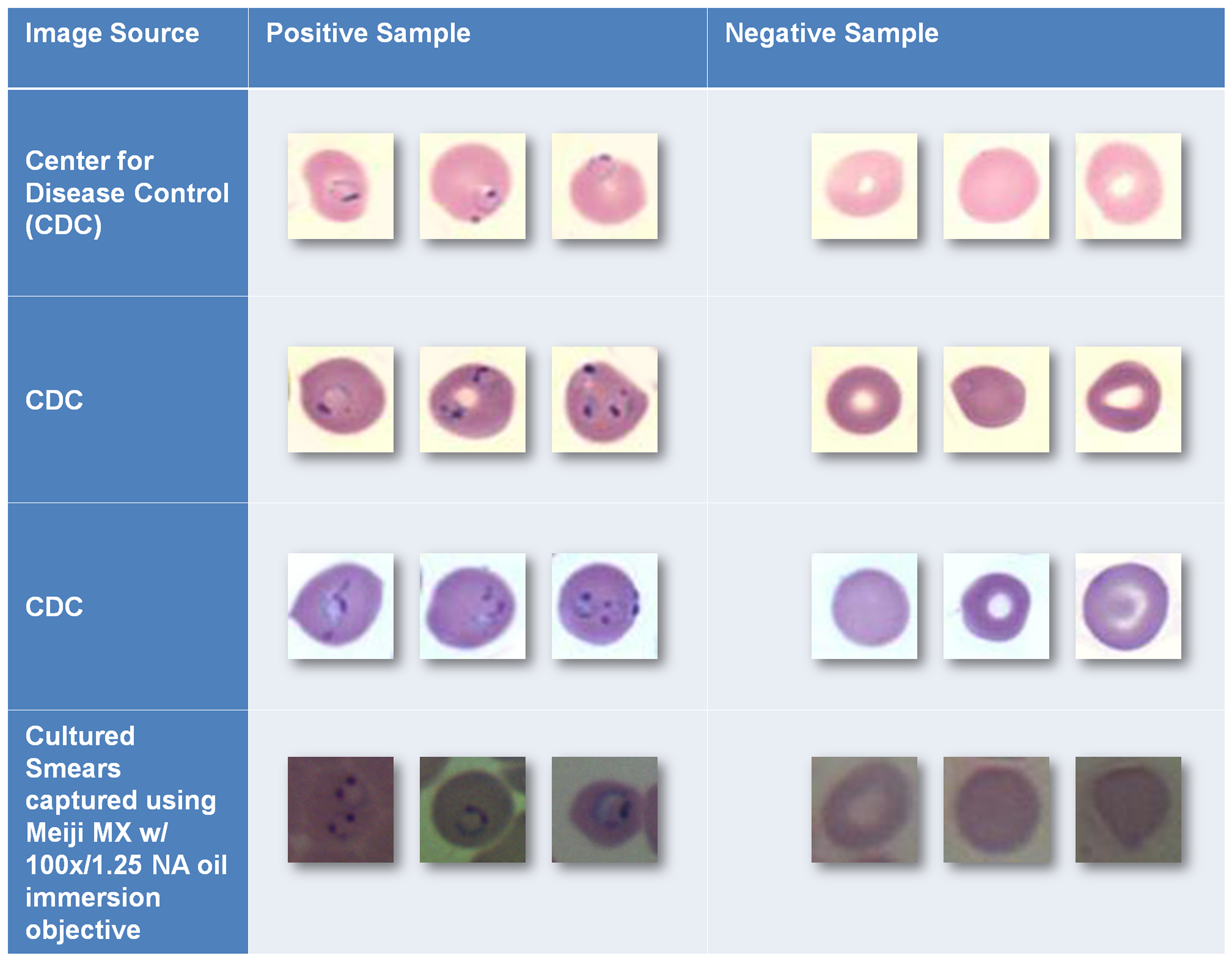

Supplement: Figure S4 — Examples of RBC images used in experiments and games. The images exhibit significantly different illumination conditions, colours and backgrounds, mimicking a real-life scenario where various different optical microscopes located at e.g., point-of-care offices and malaria clinics would be used in our games. (TIF) [file pone.0037245.s004.tif]
