# Supplementary material for: Distributed Medical Image Analysis and Diagnosis through Crowd-Sourced Games: A Malaria Case Study
Source: PLoS One. 2012 May 11;7(5):e37245. doi: 10.1371/journal.pone.0037245 (PMC3350488; doi:10.1371/journal.pone.0037245)
Supplement: Table S1 — Definition of acronyms used in the manuscript. (PDF) [file pone.0037245.s005.pdf]

Term	Acronym	Definition
True Positive	TP	Number of correctly labelled positive samples
False Positive	FP	Number of negative samples incorrectly labelled as positive
True Negative	TN	Number of correctly labelled negative samples
False Negative	FN	Number of positive samples incorrectly labelled as negative
Accuracy	ACC	$\frac{TP + TN}{TP + TN + FP + FN}$
Sensitivity or True Positive Rate	SE or TPR	$\frac{TP}{TP + FN}$
False Positive Rate	FPR	$\frac{FP}{TN + FP}$
Specificity or True Negative Rate	SP or TNR	$\frac{TN}{TN + FP}$
Positive Predictive Value	PPV	$\frac{TP}{TP + FP}$
Negative Predictive Value	NPV	$\frac{TN}{TN + FN}$
