# Supplementary material for: Distributed Medical Image Analysis and Diagnosis through Crowd-Sourced Games: A Malaria Case Study
Source: PLoS One. 2012 May 11;7(5):e37245. doi: 10.1371/journal.pone.0037245 (PMC3350488; doi:10.1371/journal.pone.0037245)
Supplement: Text S1 — This supporting text describes the mathematical and algorithmic details of the proposed framework. (PDF) [file pone.0037245.s006.pdf]

SUPPLEMENTARY TEXT

I- Definitions of acronyms used in the manuscript

The acronyms used in this manuscript are defined in Table S1.

II- Theoretical Framework for Crowd-sourced Malaria Diagnosis Game

In this section we will describe the theoretical framework with which we have approached the problem of fusing the data from multiple individuals playing the game. In doing so, we used communications and probability theory based methods.

Binary Channel Model for Gamers Diagnosing Red Blood Cells: Since we are attempting to combine decisions that are received from many gamers, we will be feeding them the same set of images to label. Therefore, there is a single sequence of images to be labelled, and each gamer will output a decision sequence. Ideally, the output of each gamer yields the correct diagnostic labels for the blood cell images. Given that each image either corresponds to a healthy cell or an infected cell, we can use binary labels to identify them: 0 for healthy and 1 for infected. Recasting our setup as a communications system, our server will act as a broadcaster of a binary sequence and each gamer will act as a *noisy Binary Channel* [1], retransmitting the symbols back along with some errors (see Figure 3 of Main Text for further details). Note that since the gamers may not necessarily make mistakes symmetrically, the probability of a gamer mislabelling a healthy cell may be different from that of mislabelling an infected cell.

System Overview: We model the framework of our games as a communications system consisting of a broadcast unit, multiple repeaters, and a receiver/decoder as shown in Figure 3 of the Main Text. In the ideal scenario, the repeaters (i.e., the gamers) would simply receive a set of incoming symbols (images to be diagnosed) from the broadcast unit, and transmit them to the receiver/decoder block, which in turn combines and stores the decisions (image labels).

Broadcast Unit - The Source of the Microscopic Images: We can model the source of our microscopic images as a broadcast unit. In this analogy, the cell images are essentially treated as binary symbols. We assume the equivalency of the two image classes infected and healthy with binary symbols 1 and 0 respectively.

The sequence of symbols $x_i, \ldots, x_N$ is broadcast to M repeater units (i.e., the gamers within the crowd) that can be viewed as M parallel noisy channels (see Figure 3 of Main Text). To be able to decode the outputs of these channels reliably, it is necessary to *learn* the channels adaptively. As such, it is crucial to embed some known symbols (i.e., *control images*) in the

output of the broadcast unit. Knowing the binary value of certain symbols/images at specific times, we can learn the conditional probabilities $p_j(y_i|x_i)$ as more symbols are transmitted by the broadcast unit and passed through the repeaters/gamers.

Additionally, an encoder unit can also be placed after the broadcast unit to increase the redundancy of the transmission, and allow for error correction at the decoder.

Gamers as Repeaters in the Communication System: Each gamer is modelled as an independent repeater that behaves as a binary channel (see Figure 3 of the Main Text). We define the error probabilities using the notation $p_j(y_i|x_i)$, corresponding to the probability that the jth user will output the symbol y_i when observing the symbol x_i (i.e. i^{th} image). In general the error probabilities are asymmetric, i.e., $p(y = x|x)$ is not the same for different values of x. However, it is difficult to accurately estimate this asymmetric probability in our games due to the imbalance in the existence of positive and negative training data (*which is true not only for malaria diagnosis but also for various biomedical image analysis/diagnosis problems such that disease signatures are relatively rare compared to healthy data*) which causes a bias towards better estimating the error probabilities when $x = 0$ (healthy case). In addition to this, another practical limitation is due to the general infeasibility of embedding large numbers of training images within the game. It is therefore more straightforward to estimate a simpler *bit-flip* probability, assuming a Binary Symmetric Channel (BSC)[1]. It was observed in our experiments that a BSC model performs better than an asymmetric model. This observation stems from the fact that there is an inherent imbalance in the number of positive and negative image samples. Since we are using a limited number of control images to estimate the probability densities of the gamers' performances, this imbalance in the control data translates to having a small number positive samples for accurate estimation of $p_{11}$and p_{10}.

Error Control Coding (ECC) in Malaria Diagnosis Games: Similar to traditional communications systems, our broadcast unit can also include an encoder to increase the information redundancy prior to transmission to the repeaters/gamers. Given that the symbols being transmitted by the broadcast unit are not known *a priori*, the only appropriate coding scheme is the *repetition code*, where each symbol is repeated for an *odd* number of times prior to transmission. At the decoder, a majority vote is taken on the channel outputs.

Leaving the communications system analogy, an ECC amounts to asking the gamer to play each image an odd number of times, and the most frequently assigned label is taken to be his or her answer for that particular image. This can also be interpreted as the gamer's confidence in the given diagnosis response. In other words, if the gamer is absolutely sure of a particular diagnosis for an image, then he or she will choose the same label on every observation. However, if the image is difficult to diagnose, then there is the chance that the gamer would not be consistent in making a decision, thus producing a lower confidence level.

Decoder for fusing Gamers' Decisions toward Malaria Diagnosis: In designing the decoder for our gaming platform, we take a *Maximum a Posteriori Probability* (MAP) approach. Suppose that we have N symbols/images $x_1, \dots, x_N$ being broadcast and relayed through $M+1$ repeaters/gamers and are received by the decoder. Also assume that we have estimates of the repeater/gamer error probabilities $p_j(y_i|x_i)$ for the jth repeater and the ith symbol. We then would like to estimate x_i given all the repeater outputs $y_i^0, \dots, y_i^M$ (see Figure 3 of the Main Text). For a particular transmitted symbol x^* we have:

$$\begin{aligned} p(x_i = x^*|\mathcal{Y}_\mathbf{i}) &= \frac{p(\mathcal{Y}_\mathbf{i}|x_i = x^*) \cdot p(x_i = x^*)}{p(\mathcal{Y}_\mathbf{i})} \\ &= \prod_{j=0}^{M} p(y_i^j|x_i = x^*) \cdot \frac{p(x_i = x^*)}{\sum_{l=0}^{1} \left[p(x_i = l) \prod_{k=0}^{M} p(y_i^k|x_i = l)\right]} \end{aligned}$$

We can then have:

$$\begin{aligned} \log p(x_i = x^*|\mathcal{Y}_\mathbf{i}) &= \sum_{j=0}^{M} \log p(y_i^j|x_i = x^*) + \log p(x_i = x^*) \\ &\quad - \log \left[\sum_{l=0}^{1} p(x_i = l) \prod_{k=0}^{M} p(y_i^k|x_i = l)\right] \end{aligned}$$

The value of z_i is taken to be x^* that maximizes the above posterior. Therefore, we have:

$$z_i = \underset{l \in \{0,1\}}{\arg\max} \left[\varphi_l + \sum_{j=0}^{M} \log p(y_i^j|x_i = l)\right] \;,\; \varphi \equiv \log p(x_i = l)$$

Automated Malaria Diagnosis using Machine Vision: In addition to the gaming and the crowd-sourcing platform described earlier, we also developed an automated machine-learning-based algorithm to detect the presence of malaria parasites (i.e., *P. falciparum*). In doing so our aim was to ultimately create a hybrid system such that machine vision and human vision can be coupled to each other to create a more efficient and accurate diagnostics platform as illustrated in Figure 4 of the Main Text.

Toward this end, instead of developing a very specific machine vision algorithm that is only applicable to malaria diagnosis we opted to create one that could potentially be also applied to

other infections that may have different visual properties. As such we developed a machine learning algorithm that extracts local colour features from cell images and feeds them into a classifier. In training the classifier, we used a small subset of the cell images in our dataset (same images that are embedded as 'control images' in the game) as the training set. We then used a variation of Adaptive Boosting [2,3] to create an ensemble of relatively weak classifiers that together produce accurate classification results as detailed in our Results section of the Main Text. For further details of our machine-learning based malaria diagnosis algorithm, please refer to the Section III of this Supplementary Text.

Hybrid (Machine + Human) Diagnosis Framework: As briefly pointed out earlier, the two discussed diagnosis methodologies (crowd-sourced gaming and machine vision) naturally lend themselves to a third hybrid diagnostics platform (see Figure 4 of the Main Text) where the images are initially processed by automated machine vision algorithms with a measure of confidence for each diagnostics decision. Images for which the machine decisions are not above a certain threshold (e.g., T) are then passed on to the human gamers to be re-diagnosed by the crowd. As demonstrated in our Experiment #4 of the Main Text, this can significantly increase the efficiency of the human gamers, since only those images that are deemed as *"difficult-to-diagnose"* by the machine vision algorithm are passed to the gamers, allowing the same number of gamers to assist in e.g., the diagnosis of more blood smears and patients per unit time.

III- Details of our Automated Recognition Algorithm for Malaria Parasites

In this manuscript our focus with regards to machine learning algorithms was to develop a general methodology such that it can also be applied to other parasites or disease signatures beyond malaria with small modifications in the visual features used for analysis. Therefore, in addition to the gaming based crowd-sourcing platform, we also developed an automated machine learning algorithm to detect the presence of malaria parasites, which helps us to compare our gaming platform's diagnosis accuracy to state-of-the-art machine vision algorithms as well as to ultimately create a hybrid system such that machine vision and human vision can be coupled to each other to create a more efficient and accurate diagnostic platform as illustrated in experiment #4 of the Main Text. In the next sub-sections we will go over some details of our algorithm.

Selection of Image Features for Recognition of Malaria Parasites: On a thin blood smear, malaria parasites are usually stained with Giemsa stain and are digitally imaged under a

brightfield microscope using a 100X oil-immersion objective lens. Giemsa stains the nuclear material in the parasite with a blue tint, and does not affect the RBC morphology.

In general, there are two possible approaches in extracting features from the acquired microscope images for the purpose of building a digital classifier. One can either attempt to extract very specific, hand-coded features or try to *learn* discriminative features from a large set of training examples. The advantage of using hard-coded features is that we can use prior knowledge of the physical/structural properties of the parasites. For example, we can look for "*ring-shaped objects*" within the RBC image as an indicator for the existence of the parasite. The advantage of using such features is that they lend themselves to *very fast* implementations and make the job of the classifier much easier. On the other hand, they are difficult to design, and are in general very inflexible to variations in sample preparation or illumination/recording conditions. For example, custom-designed malaria feature-sets that use shape and colour may not be easily modified to detect a different parasite type.

In contrast to hand-coded features, 'learned' features can be very generic and easily modified and applied to similar detection problems. They also take less time and effort to design for a particular problem, and put most of the burden of classification on the classifier itself. In this work, we preferred the second approach (i.e., learned features) and used a set of generic colour-based features for training a classifier to discriminate between RBC images that contain *P. falciparum* and those which do not.

We used two types of histogram-based features as input to our classifier. The first is a simple colour histogram of the image in grayscale. This is a feature that carries information about the general distribution of image values. We used a second, more complicated colour feature which we dubbed *Local Colour Peak Histograms* (LCPH). The LCPH for an image is formed by first generating highly quantized colour histograms in the Hue-Saturation space over local windows. For each window the two most occurring histogram bins are found. Any given pair of bins corresponds to a particular index value. In other words, an index can be assigned to the occurrence of each pair of values in the histogram, and this is the value that is recorded for each local window in the image. A second histogram is generated over the recorded indexes of all the local windows and used as the LCPH features. This feature essentially measures the relative occurrences of various colour pair co-occurrences throughout the image (see **Error! Reference source not found.**).

In addition to the above mentioned colour-based features, we used a number of more basic image features such as mean, variance, and gradient magnitude histograms to form our final feature vectors.

Classifier Training: In training our malaria classifier, we used a variation of Adaptive Boosting [2,3] and trained many weak decision-tree classifiers that together can produce a very strong classifier. In classical Adaptive Boosting, the overall classifier is tested on the complete training dataset at each iteration. Data points that are not correctly classified are then assigned larger weights for the next classifier to be trained. This is where we deviate from the classical algorithm in that instead of re-weighting the full training set and training a new classifier, we use the weights to probabilistically select a small subset of the training data for the next weak classifier to be trained. This allows for completely disjoint training data for the weak classifiers and results in very fast convergence of the boosted classifier. Refer to Algorithm S1 shown below for further details of our implementation.

Algorithm 1 Adaptive Boosting

initialize $\mathcal{D} = \{\mathbf{x}^1, y1, \ldots, \mathbf{x}^n, y_n\}, k_{max}, n_S, W_1(i) = \frac{1}{n}, i = 1, \ldots, n$

$k \leftarrow 0$

while $k < k_{max}$ **do**

  $k \leftarrow k + 1$

  train weak learner C_k using n_S samples taken from $\mathcal{D}$ according to $W_k(i)$

  $E_k \leftarrow$training error of C_k measured on $\mathcal{D}$ using $W_k(i)$

  $\alpha_k \leftarrow \frac{1}{2}\log\left[\frac{(1-E_k)}{E_k}\right]$

  $W_{k+1}(i) \leftarrow \frac{W_k(i)}{Z_k} \times \begin{cases} e^{-\alpha_k} & \text{if } h_k(\mathbf{x}^i) = y_i \text{ (correctlyclassified)} \\ e^{\alpha_k} & \text{if } h_k(\mathbf{x}^i) \neq y_i \text{ (incorrectlyclassified)} \end{cases}$

end while

return C_k and α_k for $k = 1$ to k_{max}

Algorithm S1 Summary of our Adaptive Boosting Algorithm. The total number of training points is fixed for each weak classifier, i.e., for each weak classifier a total of n_S training vectors are chosen based on the weights $W_k(i)$.

IV- Detailed analysis of gamers' performance

Detailed analysis of our largest gaming experiment (#5 - see Table 1 of the Main Text) is shown in Figures S2 and S3.

V- Sources of our Microscopic Data

The RBC images used in this work were obtained from multiple sources, which is quite important as it mimics a real-life scenario where various different optical microscopes located

at e.g., point-of-care offices and malaria clinics would be used in our gaming based diagnosis platform. The bulk of the images were captured using an in-house optical microscope with a 100X oil-immersion objective lens (numerical aperture: 1.25). Roughly 5% of the positive images and 10% of the negative images were also obtained from Center for Disease Control (CDC) database. Figure S4 shows representative images demonstrating the colour, illumination and background variability observed among these RBC images that were used in our crowd-sourced games.

References

1. Cover TM, Thomas JA (2006) *Elements of Information Theory*. Wiley.

2. Duda RO, Hart PE, Stork DG (2001) *Pattern Classification*. Wiley-Interscience.

3. Bishop CM (2006) *Pattern Recognition and Machine Learning*. Springer.

4. Papoulis A, Pillai S,U (2002) *Probability, Random Variables and Stochastic Processes*. McGraw Hill, New York.
